# Adoption of the Nutrition Care Process in Manual and Software Formats: A Systematic Review Across International Dietetic Settings

**DOI:** 10.3390/healthcare14091235

**Published:** 2026-05-03

**Authors:** Elina Polydorou, Stella A. Nicolaou, Dimitrios Papandreou, Antonis Zampelas, Eleni P. Andreou

**Affiliations:** 1 Department of Life Sciences, School of Life and Health Sciences, University of Nicosia, 46 Makedonitisas Avenue, Nicosia 1700, Cyprus; polydorou.e3@live.unic.ac.cy (E.P.); nicolaou.s@unic.ac.cy (S.A.N.); azampelas@aua.gr (A.Z.); 2 Cyprus Dietetic and Nutrition Association, P.O. Box 28823, Nicosia 2083, Cyprus; 3 Department of Clinical Nutrition & Dietetics, College of Health Sciences, University of Sharah, Sharjah 27272, United Arab Emirates; dpapandreou@sharjah.ac.ae; 4 Laboratory of Dietetics and Quality of Life, Department of Food Science and Human Nutrition, Agricultural University of Athens, 11855 Athens, Greece

**Keywords:** clinical nutrition, medical nutrition therapy, nutrition care process, implementation, electronic health records

## Abstract

**Highlights:**

**What are the main findings?**

- Barriers to NCP implementation include limited training, time constraints, and insufficient technological infrastructure, while key facilitators are professional support, peer collaboration, and access to electronic health records.
- Electronic NCP formats are generally associated with improved documentation quality, workflow efficiency, and practitioner confidence compared to manual approaches.

**What are the implications of the main findings?**

- Successful NCP implementation requires not only digital integration but also structured training, institutional support, and alignment with clinical workflows.
- Future research should prioritize high-quality interventional studies and standardized outcome measures to evaluate the impact of NCP adoption on clinical practice and patient-related outcomes.

**Abstract:**

Background/Objectives: The Nutrition Care Process (NCP) is a standardized model designed to improve the quality and consistency of nutrition care. However, its implementation remains variable across settings, influenced by factors such as time constraints, training, peer support, and technological infrastructure. This systematic review aims to synthesize the available evidence on barriers and facilitators influencing the implementation of the NCP/NCPT and to explore how different documentation formats may influence its adoption. Methods: This systematic review was conducted in accordance with PRISMA 2020 guidelines and included peer-reviewed studies published between 2009 and 2024 in English or Greek. Searches were conducted in MEDLINE, EMBASE, Scopus, CINAHL, and the Cochrane Library. Study quality was assessed using the National Heart, Lung, and Blood Institute (NIH) tool. A total of 11 reports representing eight studies were included, comprising cross-sectional, cohort, qualitative, and pilot designs. Results: The most commonly reported barriers to NCP implementation were lack of training, time constraints, and limited technological infrastructure. Key facilitators included support from national dietetic associations, peer collaboration, and access to electronic health records (EHRs). Electronic formats were more frequently described as supporting improved documentation practices, practitioner confidence, and workflow efficiency, whereas manual approaches were commonly reported as time-consuming and less structured. Conclusions: Digital integration of the NCP may support more consistent documentation practices and improved workflow processes; however, the current evidence is largely observational and heterogeneous. Evidence regarding patient-level outcomes remains limited, and definitive conclusions regarding the comparative effectiveness of implementation formats cannot be drawn. Further high-quality research is needed to evaluate the long-term clinical impact of NCP implementation.

## 1. Introduction

Variation in clinical treatment plans persists, not only between individual healthcare professionals (HCPs), but also within an individual HCP’s practice [1]. This inconsistency highlights the need for standardization in clinical care. Standardization refers to the adoption of protocols, frameworks, guidelines, and relevant terminology to ensure consistency and quality in clinical practice.

The development of clinical care processes (CCPs) dates back to the early years of medicine. However, continuous updates and the accumulation of fragmented information over time have contributed to confusion and resistance to the implementation of such processes.

In response to this need, the Academy of Nutrition and Dietetics introduced the Nutrition Care Process (NCP) in 2002 as part of an action plan to standardize nutrition care. In 2012, the NCP was incorporated as a fundamental component of the Standards of Practice (SOP) for dietitians, alongside the International Dietetics and Nutrition Terminology (IDNT), now known as the Nutrition Care Process Terminology (NCPT). Subsequently, in 2016, the NCP was included in the competency standards for qualified nutritionists and dietitians by the International Confederation of Dietetic Associations (ICDA), developed in collaboration with the European Federation of Dietetic Associations (EFAD) [2,3].

The Nutrition Care Process (NCP) was introduced by these organizations as a standardized framework to support dietitians in delivering consistent, patient-centered care. It facilitates concise and structured documentation, enhances critical thinking, and improves adherence to clinical guidelines, ultimately contributing to reduced practice variation and associated healthcare costs. Furthermore, the use of the NCP in collaborative care settings has been shown to enhance the perceived value of nutrition and dietetic professionals among other healthcare professionals (HCPs) [4].

More recently, the format through which the NCP is implemented—manual (paper-based or typed) versus electronic or software-based documentation—has emerged as a key determinant of its adoption. Differences in usability, time efficiency, integration with electronic health records (EHRs), and availability of institutional support may substantially influence implementation success across healthcare settings [4]. More recently, the format through which the NCP is implemented—manual (paper-based or typed) versus electronic or software-based documentation—has emerged as a critical determinant of adoption. In this context, manual formats refer to paper-based or non-integrated documentation methods, whereas electronic formats involve software-based or electronic health record (EHR)-integrated systems that support structured documentation. Differences in usability, time efficiency, integration with electronic health records, and institutional support may substantially influence implementation success across settings.

Despite these documented benefits, the implementation of the NCP remains inconsistent worldwide. Therefore, this systematic review aims to synthesize the available evidence on barriers and facilitators influencing the implementation of the Nutrition Care Process. In addition, it explores how different documentation formats (manual versus electronic/software-based) may influence NCP/NCPT adoption across settings. Specifically, the review seeks to identify patterns of NCP/NCPT implementation and examine the key factors influencing its uptake in clinical practice. This review is therefore positioned as an implementation-focused systematic review, aiming to understand how the Nutrition Care Process is adopted and applied in real-world clinical settings rather than to evaluate the effectiveness of specific interventions.

## 2. Materials and Methods

### 2.1. Study Selection

This systematic review was conducted in accordance with the PRISMA 2020 [5] guidelines and was prospectively registered in the International Prospective Register of Systematic Reviews (PROSPERO; Registration No: CRD420261279775). The completed PRISMA checklist is available in Supplementary File S1. The eligibility criteria were defined using the PICOS framework (Table 1).

Studies were included if they met the following criteria: published between 2009 and 2024; available in full text; written in English or Greek; original, peer-reviewed research; focused on the implementation of the Nutrition Care Process (NCP) or Nutrition Care Process Terminology (NCPT); involved registered dietitians or accredited nutrition professionals; and employed cross-sectional, cohort, pilot, or qualitative designs.

Studies were excluded if they were review articles, conference abstracts, letters, or commentaries; non-peer-reviewed publications; animal studies; focused on unofficial or developmental tools similar to the NCP; duplicates; or not directly relevant to NCP/NCPT implementation.

A systematic literature search was conducted in MEDLINE, EMBASE, Scopus, CINAHL, and the Cochrane Library, covering publications from 2009 to 2024. The final literature search was conducted in December 2024. The search strategy combined Medical Subject Headings (MeSH) and free-text terms, where applicable, related to the Nutrition Care Process and its implementation. Search terms included “Nutrition Care Process”, “NCP”, “NCPT”, “implementation”, “adoption”, “electronic health records”, and “documentation”. Boolean operators (AND, OR) were used to combine terms.

An example search strategy for MEDLINE was: (“Nutrition Care Process” OR “NCP” OR “NCPT”) AND (implementation OR adoption OR utilization) AND (electronic OR digital OR manual OR paper OR “electronic health records” OR EHR). The full search strategies for all databases are provided in Supplementary Material, Supplementary Table S2.

Two researchers independently screened titles and abstracts, followed by full-text assessment for eligibility. Discrepancies were resolved through discussion and consensus. The reference lists of included studies were also screened to identify additional relevant articles.

### 2.2. Data Extraction

Data extraction was performed independently by two researchers (E.P., E.A.) using a standardized Excel form. The following information was extracted from each included study: author(s), year of publication, country, study design, sample size, participant characteristics, implementation format of the Nutrition Care Process (manual, electronic, or mixed), and clinical or organizational setting.

In addition, key outcomes related to NCP implementation were extracted, including reported barriers and facilitators, adoption patterns, documentation practices, workflow integration, practitioner perceptions, and any reported statistical findings. Where available, information on implementation context (e.g., institutional, national, or multinational) and use of electronic health records was also recorded.

Disagreements between researchers were resolved through discussion and consensus.

Where multiple reports were derived from the same underlying study, these were identified and grouped accordingly. A mapping of reports to their corresponding studies is provided in Supplementary Table S2. To avoid double-counting, data extraction and synthesis were conducted at the study level rather than the report level.

### 2.3. Review Framework (PICOS)

The review question was structured using a modified PICOS framework (Table 1). Given the implementation-focused nature of the included evidence, the “intervention” component was interpreted as exposure/context, referring to the implementation of the Nutrition Care Process (NCP/NCPT) in clinical practice rather than a controlled intervention.

### 2.4. Quality Assessment

Study quality and risk of bias were assessed independently by two researchers (E.P., E.A.) using the National Heart, Lung, and Blood Institute (NIH) Quality Assessment Tool for Observational Cohort and Cross-Sectional Studies, a validated instrument widely used for evaluating observational study designs [6]. Each study was rated as high, medium, or low quality. Disagreements were resolved through discussion and consensus. No automation tools were used in the appraisal process.

Given the heterogeneity of the included studies and the predominance of non-comparative designs, this review was conducted as an implementation-focused synthesis rather than a conventional intervention–comparison systematic review.

Methodological quality was assessed using design-appropriate tools. The NIH Quality Assessment Tool for Observational Cohort and Cross-Sectional Studies was applied to quantitative observational studies, while qualitative studies were appraised using the Critical Appraisal Skills Programme (CASP) checklist. Critical Appraisal Skills Programme (CASP). CASP Qualitative Checklist. Available online: https://casp-uk.net/casp-tools-checklists/ (accessed on 1 December 2024). The CASP checklist was used to assess key domains including study validity, methodological rigor, clarity of data collection and analysis, and relevance of findings.

Quality assessment results were analyzed separately by study design, and no numerical aggregation was performed across heterogeneous methodologies. For observational studies, overall quality ratings were categorized as high, moderate, or low based on predefined thresholds. Quality assessment findings were interpreted within the context of study design. For quantitative observational studies, quality scores were calculated as the proportion of criteria met out of the total applicable items. Based on these scores, studies were categorized as high (≥75%), moderate (50–74%), or low (<50%) quality.

### 2.5. Data Synthesis

Given the methodological heterogeneity across the included studies, including variations in study design, clinical settings, and outcome measures, a narrative synthesis approach was employed. Study findings were summarized thematically without statistical pooling or meta-analysis. Outcomes were grouped according to implementation format (manual, electronic, or mixed) and contextual factors, including multinational, national, institutional, and prototype-based settings.

Sources of heterogeneity were explored descriptively using frequencies and proportions across these groupings. Reported *p*-values were extracted and interpreted within the context of individual studies, without transformation, imputation, or cross-study comparison.

To support interpretation, a qualitative grading of evidence was applied. Evidence was categorized as strong, moderate, or weak based on the consistency of findings, the number of supporting studies, and their methodological quality. A formal assessment of certainty (e.g., GRADE) was not feasible due to the heterogeneity of study designs and outcomes; therefore, a structured narrative approach was adopted.

**Figure 1:**
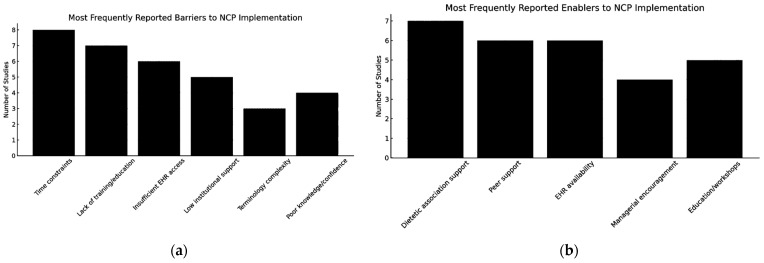
Factors influencing the Nutrition Care Process (NCP) and Nutrition Care Process Terminology (NCPT): (**a**) Most Frequently Reported Barriers to NCP Implementation; (**b**) Most Frequently Reported Enablers to NCP Implementation.

**Figure 2:**
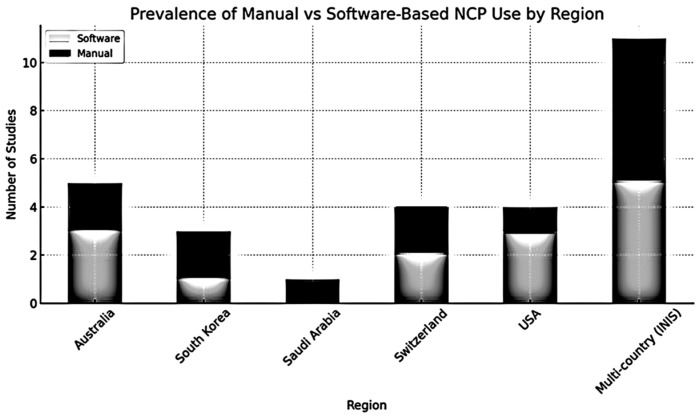
Prevalence of Manual vs. Software-Based NCP use by Region.

For the synthesis of barriers, facilitators, and implementation patterns presented in Figure 1 and Figure 2, data were aggregated at the study level to avoid duplication of evidence. Each factor was counted once per study, regardless of the number of associated reports. Frequencies therefore reflect the number of studies reporting a given factor rather than the number of individual reports or participants.

### 2.6. Protocol Registration and Amendments

After PROSPERO registration, several methodological refinements were made. First, the planned risk-of-bias assessment was revised; although Cochrane RoB tools were initially listed in the registration, the final review used the NIH Quality Assessment Tool for Observational Cohort and Cross-Sectional Studies [6], as this was considered more appropriate for the predominantly observational evidence base; qualitative studies were appraised descriptively. Second, the search strategy was refined by applying a publication-year limit of 2009–2024 and by restricting the final searches to MEDLINE, EMBASE, Scopus, CINAHL, and the Cochrane Library, with reference list screening of included studies. Third, although the registration did not specify formal comparators, the final synthesis was organized around comparison of manual and electronic/software-based NCP implementation formats. Finally, while no quantitative meta-analysis was undertaken, the final review adopted a structured narrative synthesis with grouping by implementation format and study context, together with a qualitative judgment of evidence strength. These amendments were made to better align the review methods with the nature and heterogeneity of the included studies.

### 2.7. Reporting Bias Assessment

The potential for reporting bias (including publication bias) was considered during the review process. However, due to the limited number of included studies and the methodological heterogeneity (including qualitative, cross-sectional, and mixed-methods designs), a formal quantitative assessment of reporting bias (e.g., funnel plots or statistical tests) was not feasible.

Instead, efforts were made to minimize reporting bias through a comprehensive search strategy across multiple databases, inclusion of reference list screening, and the application of predefined eligibility criteria. The potential impact of reporting bias is acknowledged as a limitation of this review.

## 3. Results

A total of 11 reports representing eight unique studies were included in this review. Where multiple reports originated from the same study, findings were synthesized at the study level to ensure that evidence was not double-counted (see Supplementary Table S2). Although studies published in Greek were included in the search strategy, no eligible studies in the Greek language met the inclusion criteria.

The most frequently reported barriers were lack of training, time constraints, and limited technological infrastructure, while key facilitators included support from national dietetic associations, peer collaboration, and access to electronic health records. Electronic formats were generally associated with improved documentation quality, enhanced workflow efficiency, and greater practitioner confidence compared to manual approaches. Despite variability in study design and setting, these findings highlight consistent themes influencing NCP adoption across diverse healthcare contexts.

**Figure 3:**
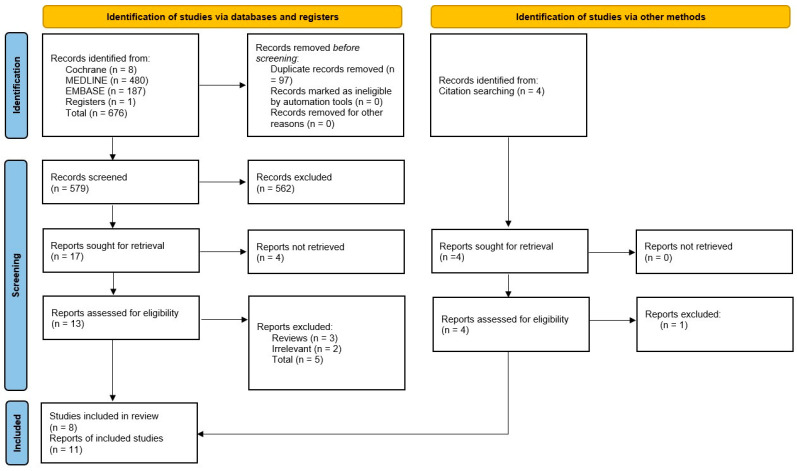
PRISMA Flow Diagram.

The PRISMA flow chart (Figure 3) summarizes the study selection process. A total of 11 reports representing eight studies were included in the final analysis. Six full-text articles were excluded after eligibility assessment. Reasons for exclusion included review article design and lack of direct relevance to NCP/NCPT implementation. These studies reflect a range of international settings and healthcare systems. Study designs included cross-sectional surveys, cohort studies, qualitative focus groups, and pilot interventions. Sample sizes ranged from 12 to 6498 participants.

### 3.1. Study Characteristics

The included studies covered diverse regions and practice environments. Data were extracted on country, study design, sample size, implementation format (manual vs. electronic), and key findings. These are summarized in Table 2.

Observed differences between countries appeared to reflect variations in implementation timelines, professional support structures, and access to technological infrastructure.

### 3.2. Outcomes and Key Findings Across Studies

The outcomes assessed across the included studies varied but primarily focused on implementation patterns, barriers and facilitators, documentation quality, and practitioner-related outcomes. A structured summary of outcomes and key findings across studies is presented in Table 3.

### 3.3. Comparison of Manual and Electronic NCP Implementation

Although no statistical synthesis was conducted, a narrative comparative analysis of manual and electronic NCP implementation formats (Table 4) suggests that electronic formats were more frequently associated with favorable outcomes. These included improved documentation practices, enhanced workflow efficiency, and greater practitioner confidence.

In contrast, manual approaches were commonly described as more time-consuming and less structured, which may contribute to lower levels of adoption. However, these observations are primarily based on non-comparative and self-reported data and should therefore be interpreted with caution.

Both formats were influenced by similar barriers, including limited training and time constraints, while facilitators such as peer support and institutional endorsement were consistently identified as important for implementation.

Overall, the findings indicate that digital integration may support improved usability and consistency; however, successful adoption of the NCP appears to depend on a combination of technological infrastructure, organizational support, and contextual factors.

### 3.4. Quality Assessment of Included Studies

Quantitative observational studies were appraised using the NIH Quality Assessment Tool for Observational Cohort and Cross-Sectional Studies. Based on predefined criteria, studies were categorized as high, moderate, or low quality. Overall, most quantitative studies were rated as moderate to high quality, with common limitations including small sample sizes and lack of blinding. The results of the quantitative quality assessment are presented in Table 5.

Qualitative studies were appraised using the Critical Appraisal Skills Programme (CASP) checklist. These studies generally demonstrated moderate to high methodological rigor; however, common limitations included limited reporting of researcher reflexivity and insufficient detail regarding data analysis procedures.

Overall, the included studies presented a moderate risk of bias. Common methodological concerns included lack of sample size justification, absence of blinding, and limited adjustment for potential confounding variables. In addition, several studies did not clearly report handling of missing data or other potential sources of bias. These limitations were more pronounced in lower-rated studies, whereas higher-quality studies demonstrated more robust methodological approaches.

### 3.5. Narrative Synthesis of Key Findings

Given the methodological heterogeneity of the included studies, a narrative synthesis approach was employed without statistical pooling. Findings were summarized descriptively across studies, and reported statistical significance was interpreted within the context of individual studies, without direct cross-study comparison.

Across the included studies, several recurring enablers of NCP implementation were identified. These included support from national dietetic associations, access to electronic health records (EHRs), and the availability of peer support or structured mentorship programs.

Commonly reported barriers included lack of formal training or education, time constraints, and limited technological infrastructure.

These findings are summarized in Figure 1. Frequencies represent the number of studies reporting each barrier or facilitator, with each study contributing once to the analysis.

### 3.6. Multinational Implementation Studies

Lövestam et al. [9] assessed the level of implementation of NCP and NCPT across 10 countries (Australia, Canada, Greece, Switzerland, Ireland, Denmark, New Zealand, the United States, Sweden, and Norway). An online, validated survey was distributed to dietitians across participating countries, where the NCP/ NCPT had been introduced at different timelines, between 2009 and 2014. The survey was composed of four modules, each of which was statistically analyzed independently. Only Module 1 (demographic characteristics, *n* = 6459) and Module 2 (NCP/NCPT implementation, *n* = 5255) were discussed in said publication. Sample characteristics differed because of the multinational concept of the study. Differences include sample size variability and the NCP/NCPT implementation strategy applied per country. Participants who reported to be unaware of the NCP were directed to the end of the study and recorded as never using nor planning to use the NCP/NCPT. Although Greece was part of the study, they had not implemented the NCP/NCPT at that time and still have not as of this writing [18]. Thus, questions related to aspects from Module 3 have been removed from the version of the questionnaire distributed to participants from Greece.

Overall, the NCP was implemented either more frequently or for longer periods than the NCPT (*p* < 0.001). The NCP and NCPT were significantly more likely to be implemented in inpatient settings (81% and 61%, respectively) and in public health settings (54% and 28%, respectively) (*p* < 0.0001).

Module 3 (factors influencing NCP implementation, *n* = 5727) was published as part of a secondary analysis investigating the attitudes, barriers and enablers related to NCP across the 10 countries [10]. A National Dietetic Association (NDA) recommendation was identified as the strongest enabler (69%), followed by peer support (63%) and the availability of electronic health records (EHR) (55%). The magnitude of an NDA recommendation demonstrated significant variation across the participating nations. While 73% of the US participants cited it as determinant, only 44% of the Norwegian dietitians did, suggesting that the preparedness and infrastructural capacity of the NDA to support such directives are crucial for effective implementation. Dietitians in Greece were not asked about the current recommendations by the NDA or about the lack of training and education relating to the NCP. Overall, the time to implement the NCP was reported as the strongest barrier (39%), followed by inadequate training and knowledge (32%). Variability was observed in the perception of the time-intensive nature of the NCP and its training demands, with 85% and 77% of Norwegian participants, respectively, citing these issues, compared with only 49% and 16% of Australian and New Zealand participants, respectively.

Data collected from Module 3 of the original study by Lövestam et al. [17], were re-analyzed by Vinci et al. [16], isolating the German and French speaking dietitians in Switzerland. The sample size was reduced to 228 dietitians, representing the 19% of the dietitians in the country in 2012 (*n* = 144 German, *n* = 84 French speaking). Awareness of the NCP/NCPT was almost universal (99%) with frequent use of the two tools by the majority (72%). Peer support and NDA influence were the strongest enablers for NCP/NCPT implementation for both groups. Barriers were similar across both groups, such as time constraints, insufficient knowledge or training, EHR and financial limitations and more. Like previous results, NDA influence varied between German and French speakers (89% vs. 77%; *p* < 0.05). Other factors with significant variation between the two groups included access to EHR (81% German vs. 44% French speakers; *p* < 0.001) and workplace NCP use enforcement (75% German vs. 53% French speakers; *p* < 0.05).

There was significant variation in the rate of implementation between each NCP and NCPT step (*p* < 0.001 for each). Country of residence was a significant factor for NCP and NCPT implementation. With the United States as a reference country, all countries excluding Australia, reported a significantly lower level of implementation (New Zealand *p* < 0.001; Canada, Switzerland, Denmark, Sweden, Norway, Ireland and Greece *p* < 0.0001). These differences likely reflect the chronological order and methods through which NCP/NCPT were introduced, with the US adopting the tools in 2003 and Australia in 2009, whereas Greece has not yet implemented them as of this writing [11,18]. Level of education did not have a significant impact on implementation (*p* = 0.253). Each additional year since completing the dietetic training was associated with a decrease in the implementation rate (*p* = 0.0001). Dietitians working with inpatients, outpatients, in teaching-academic areas and in managerial positions had the highest implementation scores (*p* < 0.0001), compared to those working in the community, research, or not currently working as dietitians. Lower associations between practice settings and implementation rates were seen in consultation and business practice (*p* = 0.007), foodservice (*p* = 0.004), and other (*p* = 0.001), with the lowest from dietitians working in public health (*p* < 0.0001).

A key limitation of the original study, and consequently of its secondary analyses, is that the United States contributed the largest proportion of the sample, which may have skewed the statistical findings. Additionally, only half of the dietitians in Greece were aware of the NCP/NCPT, potentially biasing the results. Future studies should consider more carefully selected samples, considering awareness of the NCP/NCPT as part of the inclusion criteria to enhance comparability across countries and improve generalizability.

### 3.7. National-Level Insights: Sweden, Australia and New Zealand

Vivanti, Lewis and O’Sullivan [12] assessed the knowledge about and trends around NCPT, focusing on enabling or disenabling factors and related implementation. The study took place in Australia in two phases (*n* = 218 in 2011 and *n* = 205 in 2014) and included members of the national association. The participants were asked to complete a validated survey. The time-intensive nature of NCP/NCPT was the strongest disabling factor for both surveying years (*p* = 0.84, 40% in 2011 and 41% in 2014). Although there was a significantly higher level of NCP/NCPT knowledge (mean *p* < 0.0175) and improved familiarity with and confidence in using the NCP in 2014 (*p* < 0.001) compared to 2011, the time barrier persisted.

Paradoxically, although participants in 2014 acknowledge the advantages supporting the NCP use (improved interdisciplinary communication, patient care and critical thinking (*p* < 0.001)) a higher proportion of participants, compared to 2011, still do not see clear benefits in its use. Despite the accepted benefits of NCP, there seems to be a potential disconnect between theoretical advantages and practical experience since its time-expensive process leads to implementation resistance (*p* = 0.002).

O’Sullivan, Lo and Vivanti [11], evaluated knowledge, familiarity and concerns related to NCPT as influencing factors for its implementation in the Asia-Pacific region, using the validated ASK NCP survey. The study was multinational including 377 dietitians, contributing to varying percentages of registered dietitians per locale (*n* = 209, 5% of registered professionals from Australia, *n* = 4, 7% of registered professionals from New Zealand, *n* = 51, 55% of registered professionals from Singapore, and *n* = 4 from Philippines, Indian and USA combined). Across the countries, 81.7% of the participants consider the NCP applicable to their practice, but only 65.1% currently use it. The NCP and IDNT is perceived as a useful tool for improving patient care (55%) but as inconvenient to implement (19.6%). Lack of training and support, and insufficient time, were the most reported reasons for resisting implementation of the NCP, each cited by 25.5% of respondents, followed by lack of knowledge (23.8%).

A secondary analysis of seven semi structured audiotaped focus groups (*n* = 3–8 per group) was conducted by Lövestam, Bostrom and Orrevall [13], to document the experiences and factors related to NCP/NCPT implementation of 37 dietitians, across 13 workplaces in Sweden. The participants were familiar with the NCP/NCPT, with at least one year of practicing experience. For the illustration of the results, the dietitians were categorized into three cases, based on the workforce size (i.e., Case A for small hospitals, Case B for large hospitals, Case C for solitary dietitians). Findings are documented as testimonial data rather than analytical conclusions derived from numerical data. The findings focused on assessing how the implementation strategy and support around it; peer support and group acclimatization; availability and structure of HER; and evaluation objectives influence the NCP/NCPT implementation.

Case A introduced the NCP/NCPT after the manager’s recommendation. The dietetics team saw the adaptation of NCP/NCPT as something innovative and interesting, that aligned with the diagnostic classification codes that they were already using for their EHR. Their interest to work on something new, made them oversee the difficult and time-consuming nature of NCP. Soon, the department developed their own guide with examples of problem-etiology-signs and symptoms (PES). In addition, the EHR system was formatted to include PES statements, and dietitians recommended the addition of preformatted sentences or drop-down lists. The evaluation objective for dietitians in Case A was to form a PES statement for every new patient. The goal was for 80% of those notes to include a PES statement. Peer support facilitated the acclimatization of NCP use and encouraged frequent discussions related to NCP.

Implementation of NCP/NCPT in Case B was also recommended by the manager. Dietitians that had been previously formally informed about NCP/NCPT, were designated as team leaders, leading frequent discussion meetings on NCP related topics. The evaluation goal was to write a PES statement for every new patient. Initially the NCP/NCPT was considered by many as complicated and unfit to their practice. Resistance to its implementation remained by some, as they continued to find it complex, time-intensive and ineffectual, regardless of the peer support from the leader team. At some point the discussion groups discontinued due to workforce changes that led to reduced implementation. However, during the same time, the EHR system was updated to include drop-down lists of PES statements, which made the implementation process easier and felt natural to their workflow.

The solitary dietitian represented in Case C, thought of implementing the NCP/NCPT herself, after attending some educational courses. She or her manager did not set an evaluation objective. However, for unspecified reasons she was never able to reach implementation stage. The EHR system used at her facility could not be updated to incorporate NCP related terminology. The lack of peer support, technological infrastructure and easily accessible resources made her resist the idea of implementing NCP/NCPT, as it was much faster for her to keep up with her usual handwriting notes [13].

For Cases A and B, the evaluation monitoring was discontinued for different reasons, therefore its contribution to NCP/NCPT implementation is not well understood by these findings. Incorporation of PES statements as pre-formulated sentences into the EHR for Cases A and B was a stronger facilitator compared to peer support. However, in Case C where EHR could not accommodate such pre-formulated statements, peer support could have been a strong facilitator.

A cross-sectional comparative study by Porter and Devine [17] surveyed 70 dietitians from five purposively selected hospitals, using the validated ASK NCP online survey. Two of those hospitals with at least one year of informal NCP implementation (“post-implementers”) and three without prior implementation (“pre-implementers”). No inclusion criteria applied. The survey included multiple-choice, Likert-scale, and open-ended items assessing knowledge, familiarity, confidence, support, value, barriers, training, and education related to NCP and NCPT.

Statistically, post-implementers had significantly higher knowledge scores (*p* < 0.05), greater familiarity with NCP (*p* < 0.01), greater confidence to implement (*p* < 0.01), and greater perceived support for using NCP (*p* ≤ 0.001) than pre-implementers. Overall, 96% of participants agreed that NCP/NCPT were applicable to practice, 93% valued NCP, 88% valued NCPT, 75% believed NCP/NCPT would improve patient care, and 74% felt they needed to change their practice. Among pre-implementers, 55% felt prepared to commence implementation, but 48% reported that implementation would be difficult or very difficult. Pre-implementers also reported significantly less training on NCP (*p* ≤ 0.001), although 97% indicated that further training and support would increase their confidence to implement NCP. There was no significant difference between groups regarding general concerns about implementation.

Barrier profiles differed between groups. Pre-implementers most commonly identified lack of knowledge (*n* = 25), lack of training and support (*n* = 24), limited resources (*n* = 17), time constraints (*n* = 17), concerns about decreased productivity (*n* = 16), and difficulty formulating PES statements (*n* = 14). Post-implementers also reported time constraints *(n* = 4), as well as busy workloads and part-time work status as barriers; PES statements remained challenging in some contexts (*n* = 3). Qualitative themes highlighted that successful implementation depended on allocated time for practice and regular tutorials (*n* = 8), leadership and support from managers and local NCP leaders (*n* = 6), and professional motivation through understanding the benefits of change (*n* = 5). The findings were subsequently used to justify development of an implementation package guided by Kotter’s eight-stage change model.

The previous study informed the development of an implementation package, which was later evaluated in the study by Porter, Devine and O’Sullivan [14]. They recruited a control group/ hospital (*n* = 11) and two test groups/ hospitals (*n* = 24). Three dietitians from each group were assigned as team leaders to coordinate and support the NCP/NCPT implementation. Both groups were asked to complete a validated survey pre (*n* = 35 total; *n* = 24 test groups; *n* = 11 control group) and post (*n* = 23 total; *n* = 14 test groups; *n* = 9 control group) NCP implementation, to collect information on knowledge, attitudes, and facilitating factors relating to NCP/NCPT. In addition, focus groups were conducted for the test sites after NCP implementation (*n* = 11 total, from both test groups). The dietitians from the control hospital were free to choose their implementation strategy. The dietitians from the test group received an eight-stage implementation strategy that was expected to conclude in 5 months (only seven stages were evaluated in the results). Pre- and post-implementation comparisons between the groups were insignificant for knowledge (*p* = 0.277) and familiarity (*p* = 0.804), while significantly higher for within the test groups (knowledge *p* < 0.01 and familiarity *p* = 0.025). Post implementation confidence between the groups was insignificant (*p* = 0.305), while a significant increase in confidence score, post- implementation, was observed within the test group (*p* = 0.011). Additionally, the test group was significantly more confident in using the NCPT in their practice (*p* = 0.026), they were significantly more likely to make a correct nutrition diagnosis (*p* = 0.034) and develop an appropriate PES statement (*p* = 0.034). Post-implementation perceptions within the control group, related to the applicability or value of the NCP/NCPT in their clinical practice, were significantly worse (*p* = 0.046). The time needed to complete the NCP/NCPT was similar between (*p* = 0.324) and within pre- and post-implementation group comparisons (test group *p* = 0.564; control group *p* = 0.317).

Overall, these findings suggest that exposure to NCP implementation is a stronger predictor of knowledge and confidence than the specific method used to introduce it. They also indicate that while the time allocated for adaptation may support confidence and familiarity, it does not necessarily improve the time it takes to complete the process.

### 3.8. Case Studies in Asia and the Middle East

A study conducted by Kim and Baek [8], recruited 35 clinical nutrition managers of Korean general tertiary (*n* = 24) and secondary (*n* = 11) care hospitals. Nine of those managers were from hospitals that were already implementing the NCP, while *n* = 26 from hospitals without prior implementation. Overall, 91.4% (*n* = 32; 100%/*n* = 9 from hospitals implementing, 88.5%/*n* = 23 from hospitals not implementing) were aware of the NCP/IDNT. While overall 96.9% (*n* = 31) received some sort of education/training about NCP/NCPT, their self-reported knowledge on the NCP varied (hospitals implementing/not implementing respectively: *n* = 1/*n* = 0 “very well”, *n* = 5/*n* = 3 “well”, *n* = 2/*n* = 12 “so-so”, *n* = 1/*n* = 8 “poor”). Similar trends were observed for the NCPT knowledge (hospitals implementing / not implementing respectively: *n* = 1/*n* = 0 “very well”, *n* = 4/*n* = 1 “well”, *n* = 3/*n* = 8 “so-so”, *n* = 0/*n* = 14 “poor”; *n* = 0/*n* = 1 “very poor”). The lack of knowledge is perceived as a restraining factor for NCP implementation for 66.7% (*n* = 6) of the dietitians from hospitals implementing it and as a factor to avoid implementation for 53.8% (*n* = 14) of the dietitians from hospitals not implementing it.

The NCP was described as time-intensive by 66.7% (*n* = 6) of the participants from hospitals implementing the NCP, mainly because of the changes they had to make to accommodate its implementation. Similar findings were observed from the participants from hospitals not currently implementing it, with 69.2% (*n* = 18) considering its introduction as challenging due to constraints relating to time and human resources. Benefits observed among hospital dietitians implementing the NCP included less difficulty in the decision-making process for forming a nutrition therapy plan (88.9%; *n* = 8), improved quality of nutrition therapy (77.8%; *n* = 7), more consistent performance among colleagues (55.6%, *n* = 5), and improved team communication (44.4%, *n* = 4).

A study by Alkhaldy et al. [7] included 56 dietitians across six hospitals in Jeddah, Saudi Arabia. The study had a cross-sectional design and included the questionnaire that was developed by Kim and Baek [8], with some modifications. All but one dietitian were aware of NCP. Despite the low percentage that reported to have received training on how to use the NCP (26.8%), 35.3% (*n* = 18) and 25.5% (*n* = 13) of them consider themselves to be excellent or very good users of NCP respectively, and just two to be fair users. Five dietitians were not users of the NCP. A small workforce size was the strongest barrier for NCP use (40.9%, *n* = 9). Other barriers were the lack of experience and knowledge (36.4%, *n* = 8) and the interference of NCP implementation with their usual workflow (22.7%, *n* = 5). Participants did not find the NCP to be time-consuming or difficult to complete.

### 3.9. Institutional and Prototype Evaluation

O’Sullivan [15] created an electronic prototype using the NCP format and its relative steps, with the addition of a section called “client history”. The prototype incorporated preformatted diagnoses and intervention dialogs, to choose from as appropriate. The “monitor and evaluation” step of the NCP was not tested in the pilot study due to budget constraints. The study included different methods of assessment such as questionnaires, pre- and post-statistical analysis (*n* = 12) and an audio-recorded focus group (*n* = 7) that incorporated a case study. Some of the benefits of using the NCPT, as identified by the twelve participants, were the standardization across health record notes (83%) and the systematic approach between the dietitians (75%). On the other hand, 86% of the participants identified the lack of education and training as the strongest barrier for using NCP/IDNT, followed by the time-intensive nature of the tool (14%).

When the prototype electronic health record form was adopted, confidence in using the NCP increased by 17%. Participants consistently preferred the electronic IDNT over the paper version due to its ease of use, retrospective accessibility integration with electronic health records and time efficacy. Initial barriers such as access to a computer, early-stage adjustment and security concerns were considered manageable, especially when GDPR requirements were enforced. The prototype was also considered appropriate as a training tool, with 50% of the participants reporting that it reduces training time through step-by-step menus and facilitates critical thinking. Overall, 65% of the participants are more likely to adopt the electronic IDNT, while 35% felt it did not make any difference and 8% believed it could reduce adoption.

### 3.10. Regional Patterns of NCP Format

An overview of manual versus software-based NCP use across regions is presented in Figure 2. Data are summarized at the study level, with studies categorized according to their primary reported implementation context.

Electronic formats were more frequently reported in studies conducted in countries such as the United States and Australia, whereas manual documentation was more commonly described in studies from Republic of Korea and Saudi Arabia. These observations suggest potential regional variation in NCP implementation approaches.

However, these findings are based on a limited number of heterogeneous, predominantly observational studies and should be interpreted with caution. Differences may be influenced by factors such as infrastructure, digital readiness, and health system characteristics, although these were not consistently assessed across studies.

Based on the consistency, frequency, and methodological quality of the included studies, the strength of evidence for key findings was qualitatively assessed. Strong evidence was identified for barriers such as lack of training and time constraints, as well as facilitators including support from national dietetic associations and access to electronic health records, which were consistently reported across multiple studies. Moderate evidence supported the role of peer collaboration and technological infrastructure, with some variability across settings. In contrast, evidence regarding the impact of NCP implementation on patient outcomes was limited and indirect, and therefore considered weak.

## 4. Discussion

Environmental limitations, including insufficient training and technological infrastructure constraints, were frequently reported as barriers to NCP implementation across settings. Conversely, support from national dietetic associations, peer collaboration, and access to electronic health records (EHRs) were commonly identified as key facilitators.

In studies where EHR systems were available and adaptable to incorporate the NCP, implementation was more frequently reported and was often described as being associated with improved documentation practices, increased practitioner confidence, and enhanced workflow efficiency. However, these findings are based primarily on observational and self-reported data and should be interpreted with caution. In contrast, manual or paper-based use of the NCP/NCPT was commonly described as complex and time-consuming.

Figure 1a,b summarize the most frequently reported barriers and facilitators across the included studies, based on study-level aggregation. While similar factors were reported across different geographic contexts, the limited number and heterogeneity of studies restrict the ability to draw definitive conclusions regarding their relative importance.

Regional variation in NCP format use was also observed. Studies from the United States and Australia more frequently reported the use of software-based formats, whereas studies from Republic of Korea and Saudi Arabia more commonly described reliance on manual documentation (Figure 2). These observations suggest potential contextual differences in implementation; however, they should be interpreted cautiously, as underlying factors such as infrastructure, digital readiness, and health system characteristics were not consistently assessed across studies.

The synthesis of outcomes across included studies (Table 3) highlights recurring patterns in the implementation of the Nutrition Care Process (NCP) across diverse settings. Barriers such as lack of training, time constraints, and limited technological infrastructure were frequently reported, suggesting that implementation challenges may be influenced by common systemic factors. Similarly, facilitators including support from national dietetic associations, peer collaboration, and access to electronic health records were commonly identified, underscoring the potential role of organizational and professional support structures.

Despite variability in study design and geographical context, similar findings were reported across multiple studies. However, given the predominance of observational and qualitative designs, these findings should be interpreted cautiously and are indicative of associations rather than causal relationships.

The comparison between manual and electronic NCP implementation formats (Table 4) provides further insight into potential influences on clinical practice. Electronic formats were frequently described as supporting improved documentation practices, workflow efficiency, and practitioner confidence, often attributed to structured templates and integration within electronic health record systems. In contrast, manual approaches were commonly reported as more time-intensive and less standardized.

However, these observations are largely based on non-comparative and self-reported data. Both formats were affected by similar barriers, including limited training and time constraints, suggesting that implementation is influenced by multiple contextual factors beyond format alone. While electronic formats may offer practical advantages, the current evidence remains limited and indirect, and does not support definitive conclusions regarding their superiority or impact on patient-level outcomes.

Differences observed across countries suggest that NCP implementation is strongly influenced by contextual factors, including the timing of adoption, availability of technological infrastructure, and the level of support from national dietetic associations. Countries with earlier adoption and stronger institutional support, such as the United States and Australia, demonstrated higher levels of implementation and integration of electronic formats. In contrast, lower adoption rates in other regions may reflect limited training opportunities, resource constraints, or lack of formal implementation strategies. These findings highlight the importance of context-specific approaches to NCP implementation rather than a uniform global strategy.

Given the scarcity of directly comparable studies in the nutrition and dietetics field, evidence from adjacent healthcare professions, particularly nursing, was consulted for triangulation. For example, a documentation audit study by Wang et al. [19] in the nursing context found that electronic documentation formats resulted in significantly higher completeness and comprehensiveness than paper-based formats. While causality could not be established, the structured design of electronic templates appeared to enhance both the depth and reliability of recorded data (score: 4/4 vs. 3.52/4, *p* < 0.01). Notably, nurses documented more frequently using the electronic format, which facilitated improved care planning. In contrast, dietitians often work independently, and their documentation, though critical, may not be as operationally mandatory as in nursing shifts, potentially contributing to underutilization.

Previous studies in nursing have demonstrated higher levels of compliance with standardized documentation systems, often attributed to their integration within routine clinical workflows. For example, a randomized clinical trial by Ammenwerth et al. [20] showed that computer-based nursing documentation significantly improved the legibility, completeness, and overall quality of care plans, despite a slight increase in time required due to greater documentation detail.

In contrast, dietetic practice may involve more variability in documentation requirements and less structured integration within healthcare systems. Unlike nursing, where documentation is often mandatory and embedded within routine workflows, the Nutrition Care Process (NCP) may not be consistently enforced or supported at a system level. This difference in operational context may contribute to lower and more variable adoption of the NCP across settings.

Together, these findings suggest that while electronic documentation systems may support improved quality and consistency, successful implementation of the NCP likely depends not only on technological solutions but also on stronger institutional mandates and integration into routine clinical workflows.

A more recent investigation by Tomasi et al. [21] evaluated the electronic and manual formats of the Bedside Paediatric Early Warning System (BedsidePEWS). In simulations, the electronic format led to 15.7% fewer scoring errors and improved decision-making accuracy compared to paper-based tools (*p* < 0.005).

Moreover, Snowdon et al. [22] compared the traditional and electronic Montreal Cognitive Assessment (MoCA vs. eMoCA) in a self-administered setting. While the electronic format required more time to complete (likely due to guided, audio-assisted delivery), it improved test completion and reduced errors due to skipped items—highlighting the role of digital design in enhancing data quality and patient experience.

### 4.1. Strengths and Limitations of the Included Studies

The methodological quality of the included studies was generally moderate, with only a small number meeting criteria for high-quality ratings. Common limitations included a lack of sample size justification, absence of blinding in outcome assessment, and insufficient adjustment for potential confounding variables. In several studies, the handling of missing data and other potential sources of bias were not clearly reported.

This review is also subject to several limitations. The restriction to studies published in English and Greek may have introduced language bias. Although the search strategy included Greek-language publications, no eligible studies were identified, which may indicate limited published evidence in this context or potential publication bias. Additionally, the inclusion of heterogeneous study designs, populations, and outcome measures limited the feasibility of conducting a quantitative synthesis or meta-analysis. Variability in study objectives—particularly differences between studies focusing on implementation processes versus format comparisons—may have further affected the consistency and comparability of findings.

The overall risk of bias within the included studies may have influenced the strength and reliability of the conclusions, as the predominance of observational and qualitative designs restricts the ability to establish causal relationships. Accordingly, the findings should be interpreted with caution, particularly where evidence is derived from lower-quality studies. Nevertheless, the consistency of key findings across studies of varying methodological quality provides some support for the robustness of the identified barriers and facilitators.

In addition, the possibility of reporting bias cannot be excluded, as no formal assessment (e.g., funnel plot analysis) was conducted due to the heterogeneity and limited number of included studies. Furthermore, the absence of a formal GRADE assessment may limit the interpretability of the certainty of evidence; however, a structured qualitative approach was applied to provide an overall indication of evidence strength.

### 4.2. Implications for Practice and Policy

The findings of this review highlight the need for tailored strategies to support NCP implementation in various healthcare contexts. Interventions should focus on training and education, especially in regions where formal NCP instruction is lacking.

Integrating NCP/NCPT into existing electronic health record systems (EHRs) appears to be a highly effective enabler. Therefore, healthcare administrators and national dietetic associations (NDAs) should prioritize funding and policy development to facilitate digital integration.

Additionally, promoting peer support models and standardized implementation frameworks may enhance uptake, especially in resource-limited settings or where individual dietitians work in isolation.

### 4.3. Recommendations for Future Research

Future research should explore the longitudinal impact of NCP/NCPT implementation on patient-related outcomes, beyond implementation metrics such as documentation quality or practitioner satisfaction.

There is a need for high-quality interventional studies, including randomized controlled trials (RCTs) and controlled before–after studies, to evaluate the effectiveness of NCP training programs, electronic health record (EHR) integration, and policy-level interventions.

Further research is also required in underrepresented regions, including Eastern Europe, the Middle East, and parts of Asia and Africa, to better understand context-specific barriers and facilitators within diverse healthcare systems.

## 5. Conclusions

This systematic review highlights the potential value of standardized clinical frameworks, such as the Nutrition Care Process (NCP) and Nutrition Care Process Terminology (NCPT), in supporting consistency and structure in dietetic practice. The findings suggest that implementation of these tools may contribute to improved documentation practices and more standardized approaches to patient care; however, the strength of this evidence is limited by the predominantly observational and heterogeneous nature of the included studies.

Across diverse healthcare settings, electronic integration of the NCP within electronic health records (EHRs) was frequently reported as a facilitating factor for implementation, particularly when combined with institutional support and professional training. Conversely, manual or paper-based approaches were often described as more challenging to implement. However, these observations are largely based on non-comparative and self-reported data, and should therefore be interpreted with caution.

Overall, the evidence indicates that digital formats may offer practical advantages in supporting NCP adoption, but the current literature remains limited, context-dependent, and lacks robust comparative and patient-level outcome data. As such, definitive conclusions regarding the superiority of electronic over manual formats cannot be drawn.

Future efforts should focus on strengthening the evidence base through well-designed comparative and implementation studies, as well as on developing context-sensitive strategies, including training, institutional support, and appropriate digital infrastructure.

In conclusion, while this review provides a global overview of barriers and facilitators to NCP implementation, further high-quality research is required to better understand how different implementation approaches influence clinical practice and patient outcomes.

## Figures and Tables

**Table 1:** PICOS framework used in this systematic review.

Component	Description
P (Population)	Registered Dietitians, nutritionists, and healthcare professionals involved in the implementation of the Nutrition Care Process
(I)Exposure/Context	Implementation of the Nutrition Care Process (NCP/NCPT) in clinical practice
C (Comparison)	Not restricted; where available, differences between manual (paper-based or typed) and electronic/software-based formats were considered
O (Outcomes)	Barriers and facilitators to implementation, adoption patterns, usability, documentation quality, workflow integration, and related outcomes
S (Study Design)	Observational studies, qualitative studies, mixed-methods studies, and implementation reports

**Table 2:** Summary of study characteristics of the included reports. Study IDs indicate grouping of reports derived from the same underlying study (Supplementary Table S2).

Authors	Study ID	Country	Study Design	Sample Size	NCP-Format	Key Findings	Quality *
Alkhaldy et al. [7]	Study A	Saudi Arabia	Cross-sectional (quantitative)	56 dietitians	Manual (Excel use; some informal digital methods)	Majority were aware of NCP but lacked formal training; barriers included lack of experience, inadequate staffing, and protocol conflicts.	Medium
Kim and Baek [8]	Study B	Republic of Korea	Questionnaire survey	35 hospitals	Mixed (Manual and Computerized form)	Limited full NCP implementation; 21 hospitals plan future adoption	Medium
Lövestam et al. [9]	Study C	10 countries	International cohort (survey)	6498 dietitians	Mostly manual; NCPT less used	NCP used more than NCPT (*p* < 0.001); stronger implementation in inpatient settings; Greece lowest awareness (50%)	High
Lövestam et al. [10]	Study C	10 countries	Cross-sectional (validated online survey)	5727 professionals	Mixed	Top barrier: time (39%); top enabler: national dietetic association support (69%); country-specific differences significant	High
O’Sullivan, Lo and Vivanti [11]	Study D	Australia, New Zealand, Singapore	Online survey	377 dietitians	Mixed	65% currently use NCP; main barriers: lack of time, training, knowledge, and EHR access; benefit to patient care statistically supported	High
Vivanti, Lewis and O’Sullivan [12]	Study D	Australia	Cohort design (2011 and 2014)	218 in 2011, 205 in 2014	Online survey using ASK NCP	Knowledge barrier reduced (*p* < 0.001); improved perceptions of NCP structure, documentation clarity, and care quality	High
Lövestam, Bostrom and Orrevall [13]	Study E	Sweden	Qualitative (focus groups)	37 dietitians	Manual, semi-guided	Independent dietitians struggled without formal support; time pressure and terminology complexity were major issues	Medium
Porter, Devine and O’Sullivan [14]	Study F	Australia	Mixed-methods (with control group)	35 dietitians	Manual vs. software and training	Training package increased knowledge, support, and familiarity; barriers: unclear implementation strategy, PES difficulty	High
O’Sullivan [15]	Study G	Australia	Pilot study (survey, case study, pre-/posttest)	12 dietitians	Electronic prototype	86% had not used NCP/NCPT due to knowledge gaps; prototype improved speed, accessibility, and documentation clarity	Medium
Vinci et al. [16]	Study H	Switzerland	Qualitative (secondary analysis)	228 dietitians	Mixed	99% awareness; key enablers: peer support, EHR, association recommendations; language-based differences observed	High
Porter et al. [17]	Study F	Australia	Mixed-methods (with control)	70 dietitians	Online survey using ASK NCP	Post implementers of NCP report higher knowledge, familiarity, confidence, with main barriers being workload, time constraints. Pre- implementers reported lack of knowledge, training and support as barriers.	High

Note: Quality scores (%) were calculated as the proportion of satisfied criteria out of the total applicable items. * Quality ratings were defined as high (≥75%), moderate (50–74%), and low (<50%).

**Table 3:** Summary of outcomes and key findings across included studies.

Study	Study Design	NCP Format	Outcomes Assessed	Key Findings
**Alkhaldy et al. [7]**	Cross-sectional	Manual	Awareness, barriers, training	Majority aware but lacked training; barriers included lack of experience, staffing, and workflow conflicts
**Kim and Baek [8]**	Survey	Mixed	Knowledge, implementation, barriers	Limited implementation; knowledge gaps and training needs identified
**Lövestam et al. [9]**	Internation-al sur-vey/cohort	Mostly manual	Implementation rates, barriers, facilitators	NCP more used than NCPT; stronger implementation in inpatient settings; NDA support and EHR access key facilitators
**Lövestam et al. [10]**	Cross-sectional survey	Mixed	Barriers, facilitators	Time constraints most common barrier; NDA support strongest facilitator
**O’Sullivan, Lo and Vivanti [11]**	Survey	Mixed	Adoption, barriers	Moderate adoption; barriers included lack of time, training, and EHR access
**Vivanti, Lewis and O’Sullivan [12]**	Cohort sur-vey	Mixed	Knowledge, perceptions, implementation	Improved knowledge and confidence over time, but time barrier persisted
**Lövestam, Bostrom and Or-revall [13]**	Qualitative (focus groups)	Manual	Implementation experiences	Time pressure, lack of support, and terminology complexity hindered implementation
**Porter, Devine and O’Sulli-van [14]**	Mixed-methods	Manual vs. electronic (training)	Knowledge, confidence, implementation	Training improved knowledge and confidence; implementation strategy influenced outcomes
**O’Sullivan [15]**	Pilot study	Electronic	Usability, documentation, adoption	Electronic prototype improved speed, accessibility, and documentation clarity
**Vinci et al. [16]**	Secondary analysis	Electronic/Mixed	Implementation, facilitators	High awareness; peer support and EHR key facilitators
**Porter et al. [17]**	Intervention development	Mixed	Implementation support	Structured implementation improved familiarity but not time efficiency

NCP = Nutrition Care Process; NCPT = Nutrition Care Process Terminology; NDA = Nutrition and Dietetics Association; EHR = Electronic Health Records.

**Table 4:** Comparison of manual versus electronic NCP implementation across included studies.

Outcome	Manual Format	Electronic Format	Evidence Summary
**Implementation rate**	Lower adoption; inconsistent use	Higher adoption when integrated into EHRs	Higher adoption of electronic formats may be supported by integration within existing clinical systems, reducing duplication of work and facilitating routine use
**Time efficiency**	Often perceived as time-consuming	Improved efficiency, though initial adaptation required	Electronic formats may enhance efficiency through structured templates and automated documentation processes, although initial training and adaptation are required
**Documentation quality**	Variable, less structured	More standardized, complete, and legible	Digital systems support standardized documentation through predefined fields, embedded terminology (NCPT), and improved legibility
**Practitioner confidence**	Lower confidence due to complexity and lack of support	Increased confidence with structured digital tools	Structured interfaces, decision-support features, and guided workflows may enhance practitioner confidence and reduce uncertainty
**Training requirements**	High reliance on individual knowledge and experience	Facilitated through structured systems and digital tools	Electronic systems may reduce reliance on individual expertise by embedding guidance and standardized processes, although training remains necessary
**Workflow integration**	Often perceived as disruptive	Better integration within clinical workflows when embedded in EHRs	Integration within EHR systems may support smoother workflow alignment and reduce fragmentation of documentation processes
**Barriers**	Time constraints, lack of training, terminology complexity	Initial technical challenges, access to systems	Both formats are affected by common barriers; however, electronic formats introduce additional technical and system-related challenges
**Facilitators**	Peer support, institutional encouragement	EHR access, structured templates, NDA support	Electronic systems may enhance existing facilitators by providing structural support, although organizational and professional factors remain critical

NCP = Nutrition Care Process; NCPT = Nutrition Care Process Terminology; EHR = Electronic Health Records; NDA = Nutrition and Dietetics Association.

**Table 5:** Quality assessment of quantitative observational studies (NIH Tool).

Authors (Ref)	YES	NO	NR	NA	Total Applicable	Quality Score (%)	Quality Rating
**Alkhaldy et al. [7]**	7	6	1	0	14	50.0	Moderate
**Kim and Baek [8]**	8	5	1	0	14	57.1	Moderate
**Lövestam, Steiber, et al. [9]**	12	2	0	0	14	85.7	High
**Lövestam, Vivanti et al. [10]**	8	5	1	0	14	57.1	Moderate
**O’Sullivan, Lo and Vivanti [11]**	8	4	2	0	14	57.1	Moderate
**Vivanti, Lewis and O’Sullivan [12]**	10	3	0	1	13	76.9	High
**O’Sullivan [15]**	7	4	1	0	12	58.3	Moderate
**Porter et al. [17]**	9	4	1	0	14	64.3	Moderate

Note: YES = criterion satisfied; NO = criterion not satisfied; NR = not reported; NA = not applicable. Total applicable refers to the number of criteria relevant to each study. Quality scores (%) were calculated as the proportion of satisfied criteria out of the total applicable items. Studies were categorized as high (≥75%), moderate (50–74%), or low (<50%) quality based on these scores. The NIH Quality Assessment Tool was applied only to quantitative observational studies.

## Data Availability

Data extracted from the included studies and supporting materials are available within the article and its Supplementary Materials (Table S2).

## Supplementary Materials

The following supporting information can be downloaded at: https://www.mdpi.com/article/10.3390/healthcare14091235/s1. Table S1: Completed PRISMA 2020 checklist; Table S2: Full search strategies for all databases used in the systematic review.

## Abbreviations

The following abbreviations are used in this manuscript:

CCP(s)	Clinical Care Process(es)
EHR	Electronic health record
EMBASE	Excerpta medica database
IDNT	International dietetics and nutrition terminology
MEDLINE	Medical literature analysis and retrieval system online
NCP	Nutrition Care Process
NCPT	Nutrition Care Process Terminology
NDA	National dietetic association
NIH	National heart, lung, and blood institute
PES	Problem, etiology, signs and symptoms
PICOS	Participants, interventions, comparison, outcomes, study design
PRISMA	Preferred reporting items for systematic reviews and meta-analyses
